# Supplementary material for: Physiological and life history changes associated with seasonal adaptation in the cactophilic Drosophila mojavensis
Source: Biol Open. 2022 Oct 26;11(10):bio059610. doi: 10.1242/bio.059610 (PMC9637388; doi:10.1242/bio.059610)
Supplement: Supplementary information [file biolopen-11-059610-s1.pdf]

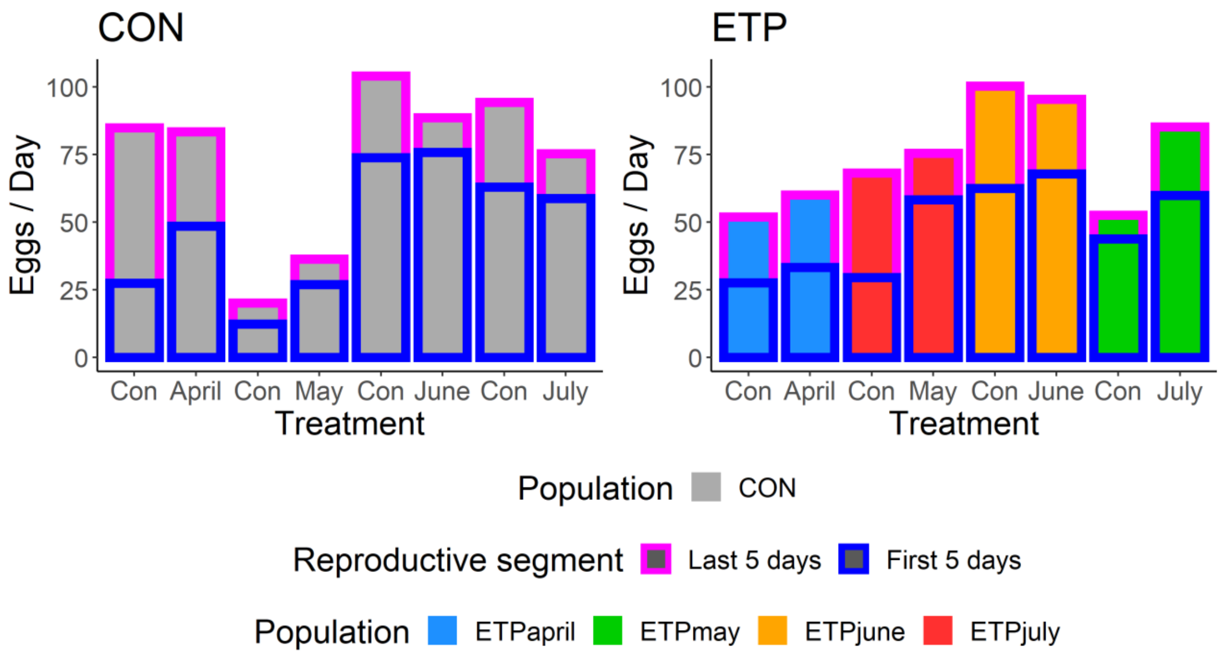

Fig. S1. Mean fecundity measurements (eggs/day per female). The average number of eggs laid per day for the first five days is outlined in dark blue, while the average number of eggs laid per day for the last five days is outlined in pink. See tables S4 and S5 for additional information on subgroup differences.

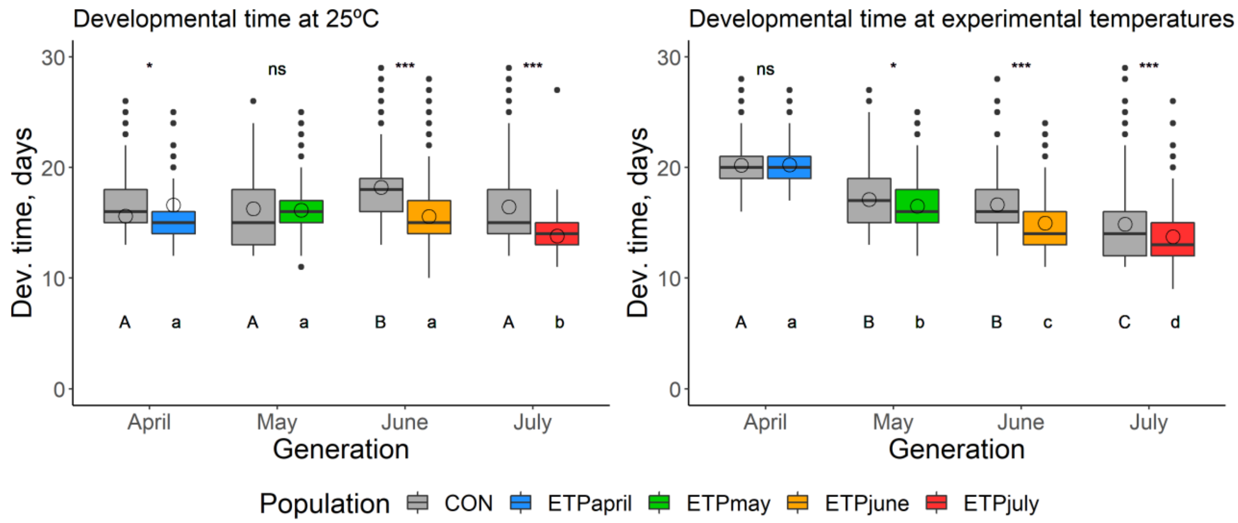

Fig. S2. Developmental time measured as the number of days from oviposition to adult eclosion. Box plot horizontal lines indicate the first, second, and third quartiles and whiskers show the extreme upper and lower values within 1.5 times the interquartile range (IQR). Solid circles are values beyond 1.5× IQR. Open circles denote the average for the group. Asterisks indicates significance (*p < = 0.05, **p < = 0.01, ***p < = 0.001) between CON and ETP populations of the same generation. Uppercase letters indicate significant differences across generations of the CON population, while lowercase letters indicate significant differences across generations of the ETP.

Biology Open • Supplementary information

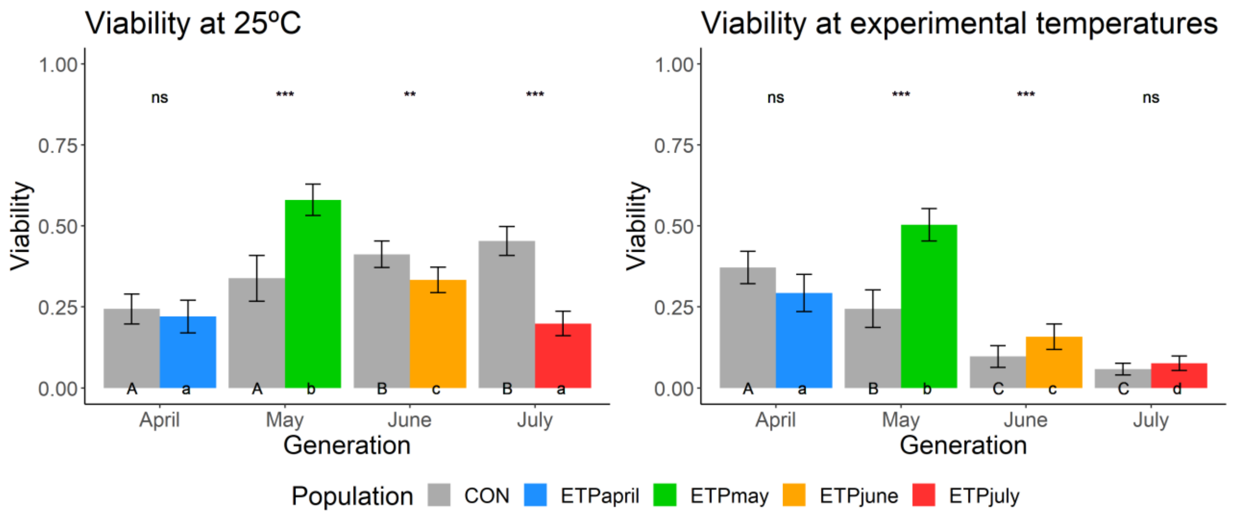

Fig. S3. Mean egg-to-adult viability (± std. error) across the populations and treatments. Asterisks indicates significance (*p < = 0.05, **p < = 0.01, ***p < = 0.001) between CON and ETP populations of the same generation. Uppercase letters indicate significant differences across generations of the CON population, while lowercase letters indicate significant differences across generations of the ETP.

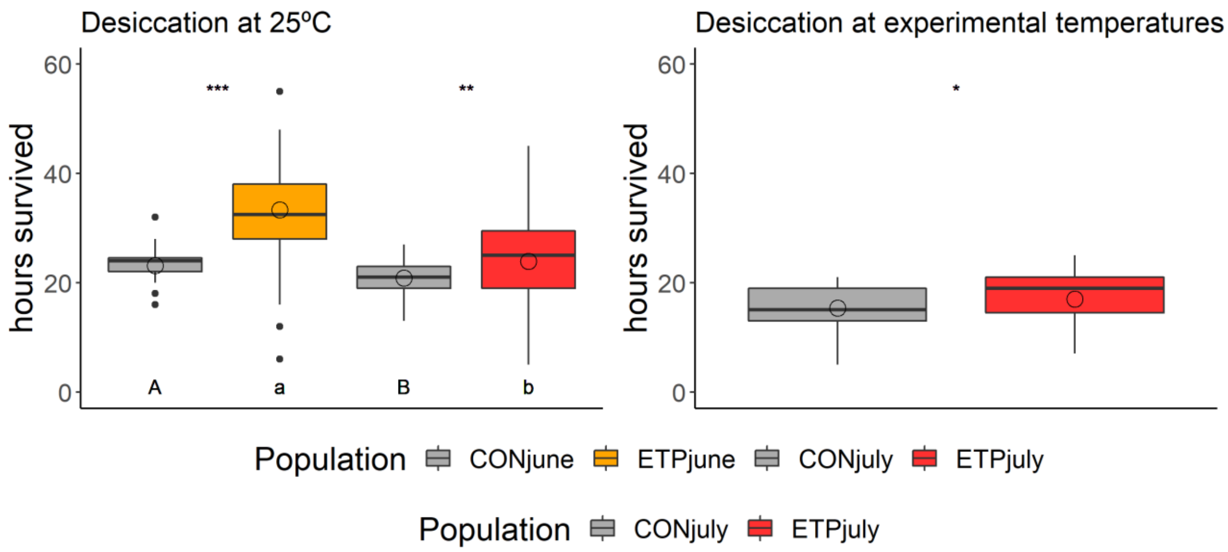

Fig. S4. Desiccation (hours) resistance for the June and July generations. Box plot horizontal lines indicate the first, second, and third quartiles and whiskers show the extreme upper and lower values within 1.5 times the interquartile range (IQR). Solid circles are values beyond 1.5× IQR. Open circles denote the average for the group. Asterisks indicates significance (*p < = 0.05, **p < = 0.01, ***p < = 0.001) between CON and ETP populations of the same generation. Uppercase letters indicate significant differences across generations of the CON population, while lowercase letters indicate significant differences across generations of the ETP.

Biology Open • Supplementary information

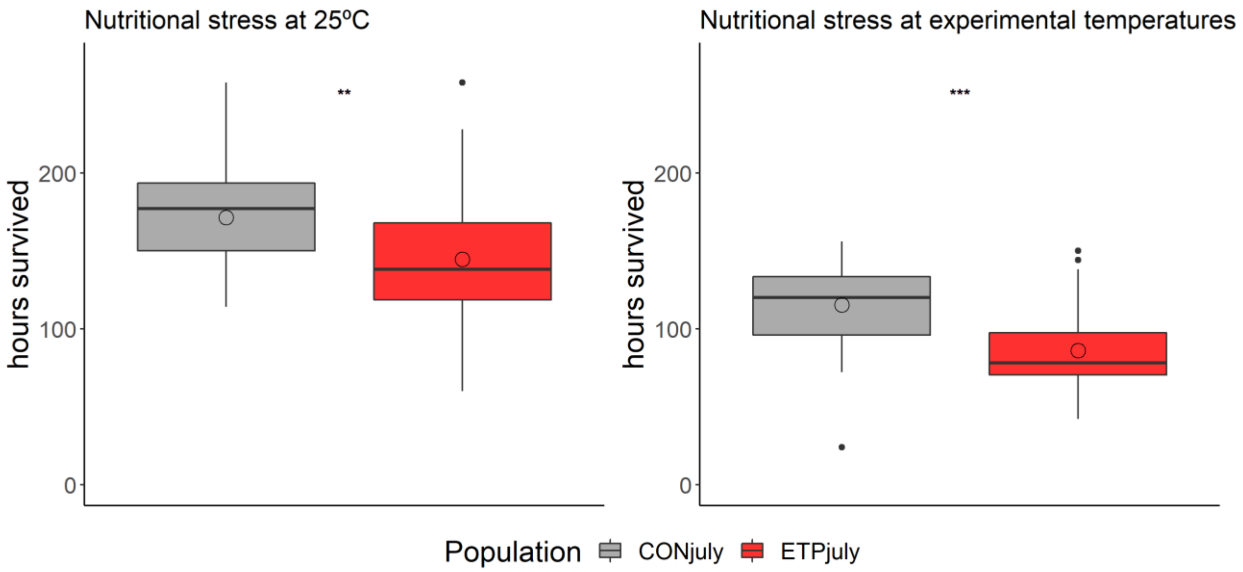

Fig. S5. Nutritional stress survival (hours) for the July generation. Box plot horizontal lines indicate the first, second, and third quartiles and whiskers show the extreme upper and lower values within 1.5 times the interquartile range (IQR). Solid circles are values beyond 1.5× IQR. Open circles denote the average for the group. Asterisks indicates significance (*p < = 0.05, **p < = 0.01, ***p < = 0.001) between CON and ETP populations of the same generation.

Biology Open • Supplementary information

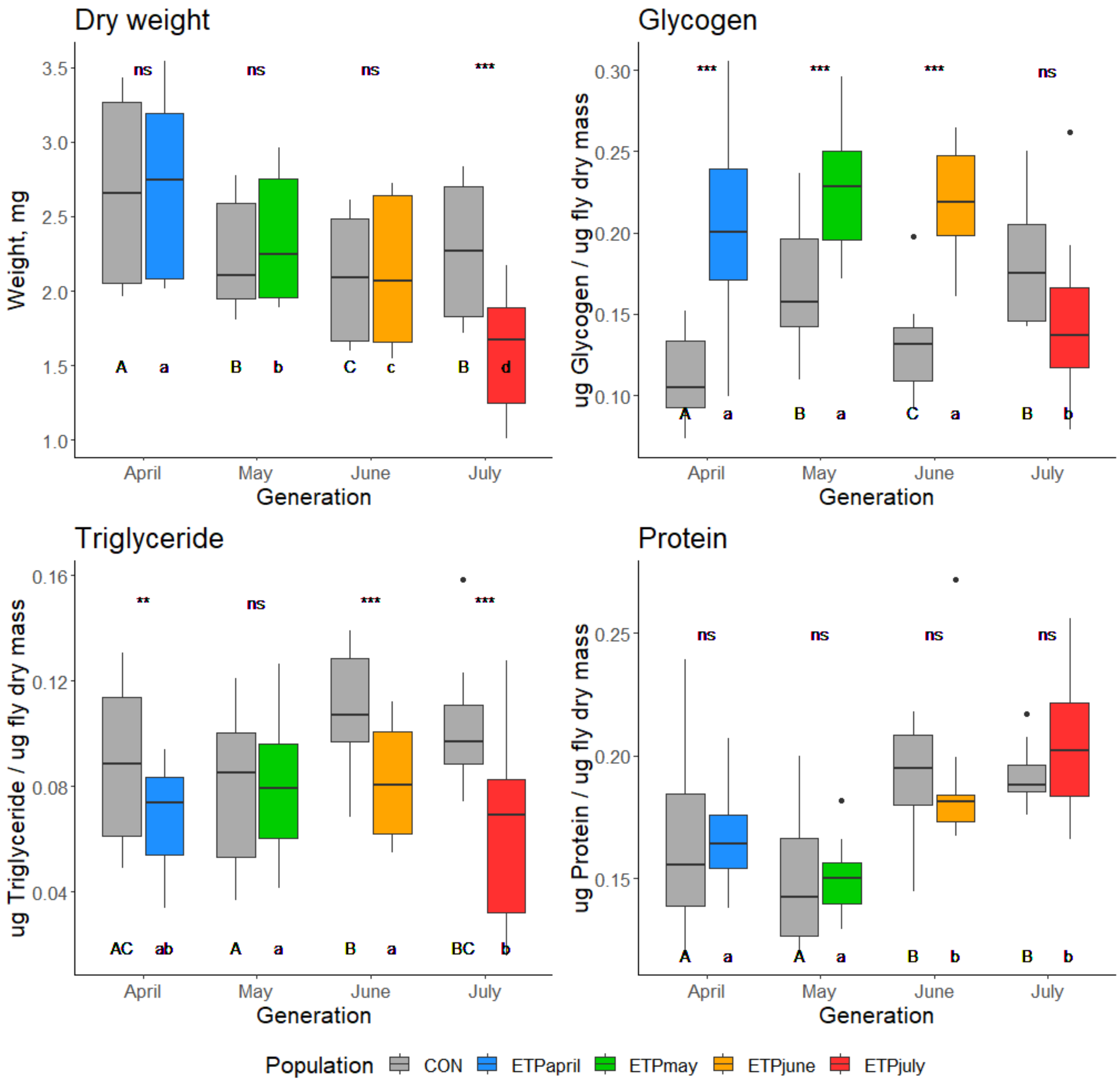

Fig. S6. (A) Dry weight (mg) (B) Glycogen content (µg glycogen / µg fly dry mass) (C) Triglyceride content (µg triglycerides / µg fly dry mass) (D) Protein content (µg protein / µg fly dry mass). Box plot horizontal lines indicate the first, second, and third quartiles and whiskers show the extreme upper and lower values within 1.5 times the interquartile range (IQR). Solid circles are values beyond 1.5× IQR. Asterisks indicates significance (*p < = 0.05, **p < = 0.01, ***p < = 0.001) between CON and ETP populations of the same generation. Uppercase letters indicate significant differences across generations of the CON population, while lowercase letters indicate significant differences across generations of the ETP.

Biology Open • Supplementary information

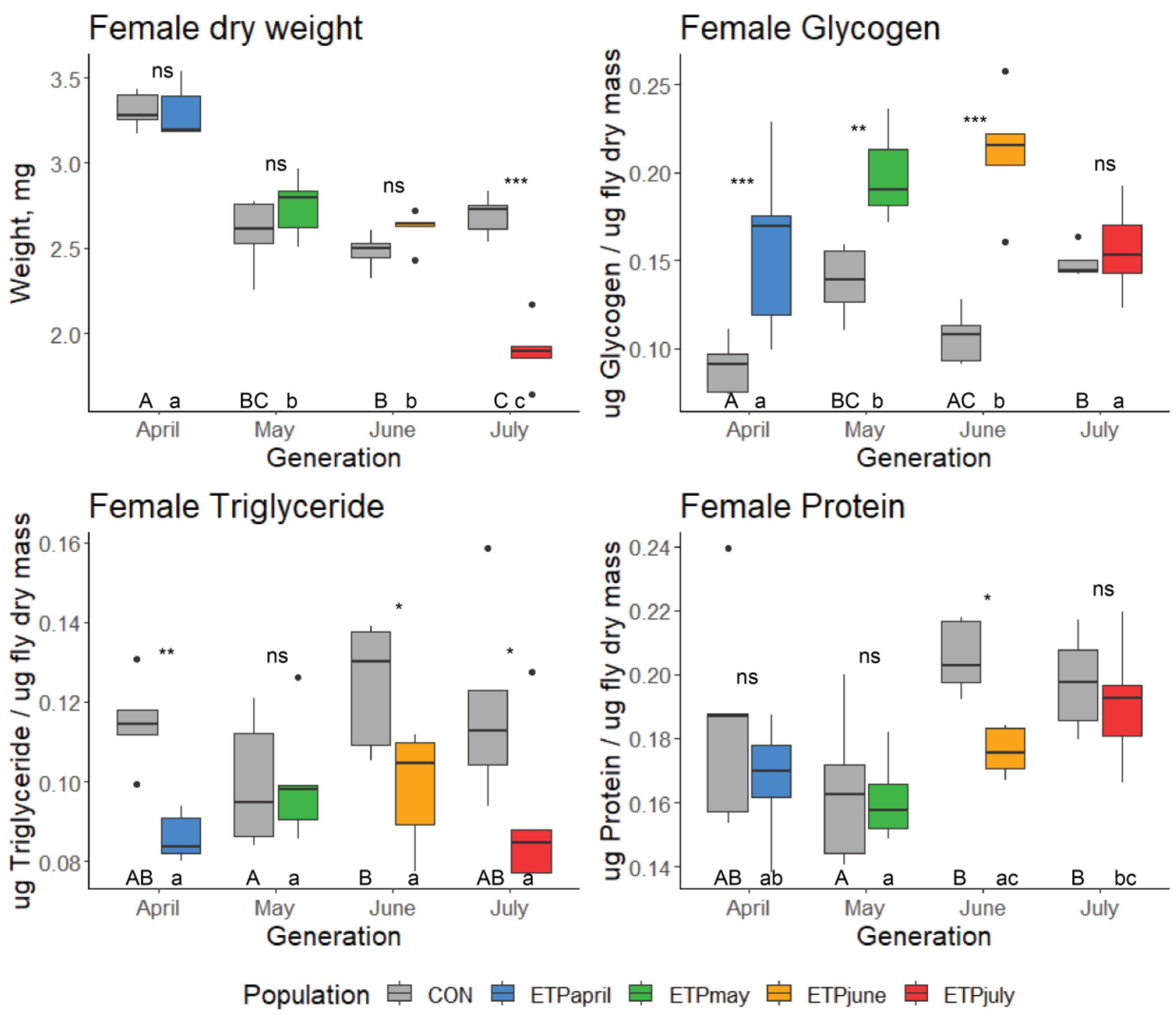

Fig. S7. Female Dry weight (mg), Female Glycogen content (μg glycogen / μg fly dry mass), Female Triglyceride content (μg triglycerides / μg fly dry mass), and Female Protein content (μg protein / μg fly dry mass). Box plot horizontal lines indicate the first, second, and third quartiles and whiskers show the extreme upper and lower values within 1.5 times the interquartile range (IQR). Solid circles are values beyond 1.5× IQR. Asterisks indicates significance (*p < = 0.05, **p < = 0.01, ***p < = 0.001) between CON and ETP populations of the same generation. Uppercase letters indicate significant differences across generations of the CON population, while lowercase letters indicate significant differences across generations of the ETP.

Biology Open • Supplementary information

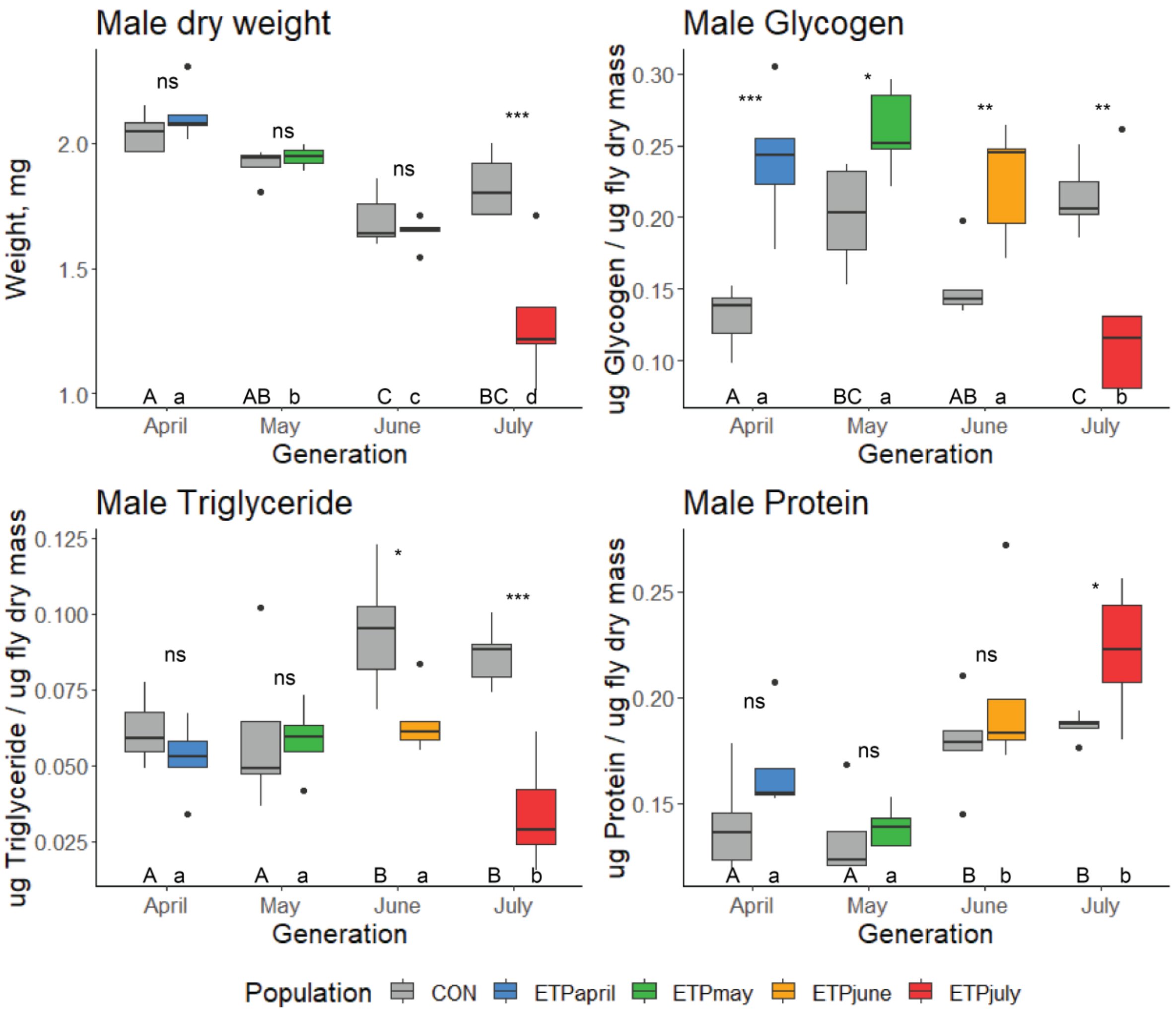

Fig. S8. Male Dry weight (mg), Male Glycogen content (µg glycogen / µg fly dry mass), Male Triglyceride content (µg triglycerides / µg fly dry mass), and Male Protein content (µg protein / µg fly dry mass). Box plot horizontal lines indicate the first, second, and third quartiles and whiskers show the extreme upper and lower values within 1.5 times the interquartile range (IQR). Solid circles are values beyond 1.5× IQR. Asterisks indicates significance (*p < = 0.05, **p < = 0.01, ***p < = 0.001) between CON and ETP populations of the same generation. Uppercase letters indicate significant differences across generations of the CON population, while lowercase letters indicate significant differences across generations of the ETP.

Biology Open • Supplementary information

Table S1. A. Experimental Temperature Cycles

Hour	April (˚C)	May (˚C)	June (˚C)	July (˚C)
0	17.5	20.3	24.7	28.4
1	16.1	18.7	23.4	27.7
2	14.9	17.8	22.4	27.3
3	14.1	16.9	21.4	26.5
4	13.4	16.2	20.5	25.9
5	12.7	15.5	20.2	25.4
6	12.2	15.7	21.0	25.4
7	15.5	20.7	26.1	27.5
8	19.1	23.6	29.1	29.2
9	21.7	26.0	31.5	30.9
10	23.7	28.0	33.4	32.6
11	25.2	29.4	34.9	33.9
12	26.4	30.4	36.1	35.1
13	27.1	31.3	36.8	35.9
14	27.8	31.7	37.2	36.4
15	28.1	31.8	37.5	36.6
16	27.9	31.7	37.1	36.3
17	27.4	30.9	36.5	35.7
18	26.3	29.9	35.4	34.6
19	24.7	28.0	33.8	33.1
20	23.1	26.4	32.0	31.9
21	21.7	25.0	30.7	31.1
22	20.0	23.6	28.8	30.4
23	18.7	21.6	26.8	29.3

B. Light Cycles, given in 24hr format

	April	May	June	July	Control
Light On	06:00	05:30	05:25	05:35	07:00
Light Off	09:00	09:45	09:40	09:40	09:00
Hours of light	13h	14h 15min	14h 15min	14h 5min	12h

Biology Open • Supplementary information

Table S2. Adult activity ANOVA *horizontal* test

**ETP**	**Chisq**	**Df**	**Pr(>Chisq)**
Developmental	75.209	3	3.268e-16
Sex	0.792	1	0.373
Developmental:Sex	7.114	3	0.068
**CON**	**Chisq**	**Df**	**Pr(>Chisq)**
Developmental	1.618	3	0.655
Sex	1.0626	1	0.302
Developmental:Sex	13.063	3	0.004

vertical test.

**April**	**Chisq**	**Df**	**Pr(>Chisq)**
Developmental	.954	1	0.328
Test	9.593	1	0.001
Sex	1.136	1	0.286
Developmental:Test	0.113	1	0.737
Developmental:Sex	14.643	1	0.000
Test:Sex	1.626	1	0.202
Developmental:Test:Sex	8.723	1	0.003
**May**	**Chisq**	**Df**	**Pr(>Chisq)**
Developmental	0.342	1	0.558
Test	38.41	1	5.733e-10
Sex	0.499	1	0.480
Developmental:Test	13.938	1	0.001
Developmental:Sex	9.337	1	0.002
Test:Sex	0.147	1	0.702
Developmental:Test:Sex	13.816	1	0.001
**June**	**Chisq**	**Df**	**Pr(>Chisq)**
Developmental	7.915	1	0.005
Test	22.647	1	1.946e-06
Sex	0.021	1	0.884
Developmental:Test	4.685	1	0.030
Developmental:Sex	0.955	1	0.331
Test:Sex	0.311	1	0.577
Developmental:Test:Sex	0.062	1	0.804
**July**	**Chisq**	**Df**	**Pr(>Chisq)**
Developmental	2.893	1	0.089
Test	14.578	1	0.001
Sex	3.23	1	0.072
Developmental:Test	0.793	1	0.373
Developmental:Sex	2.844	1	0.092
Test:Sex	0.0001	1	0.992
Developmental:Test:Sex	3.601	1	0.058

Biology Open • Supplementary information

Table S3. Adult activity *post hoc* tests

Adult activity ETP *horizontal post hoc* test. P- values shown.

ETP	June	July	May
April	0.000	<.0001	0.003
June		<.0001	<.0001
July			0.079

Activity CON *horizontal post hoc* test. P- values shown.

CON	June Female	July Female	May Female	April Male	June Male	July Male	May Male
April Female	0.003	0.087	0.068	0.002	0.086	0.040	0.218
June Female		0.218	0.265	0.448	0.556	0.806	0.282
July Female			0.905	0.085	0.703	0.469	0.914
May Female				0.103	0.773	0.528	0.840
April Male					0.086	0.201	0.019
June Male						0.662	0.534
July Male							0.290

Biology Open • Supplementary information

Table S4. Fecundity ANOVA *horizontal* test.

**ETP *horizontal***	**Chisq**	**Df**	**Pr(>Chisq)**
Developmental	122.34	3	< 2.2e-16
early_late	1163.32	1	< 2.2e-16
Developmental:early_late	513.92	3	< 2.2e-16
**CON *horizontal***	**Chisq**	**Df**	**Pr(>Chisq)**
Developmental	122.34	3	< 2.2e-16
early_late	1163.32	1	< 2.2e-16
Developmental:early_late	513.92	3	< 2.2e-16

vertical test.

**April**	**Chisq**	**Df**	**Pr(>Chisq)**
Developmental	382.679	1	< 2.2e-16
Test	4.812	1	0.028
early_late	40.438	1	2.030e-10
Developmental:Test	0.417	1	0.517
Developmental:early_late	20.588	1	5.693e-06
Test:early_late	98.285	1	< 2.2e-16
Developmental:Test:early_late	165.738	1	<2.2e-16
**May**	**Chisq**	**Df**	**Pr(>Chisq)**
Developmental	950.427	1	< 2.2e-16
Test	69.132	1	< 2.2e-16
early_late	268.116	1	< 2.2e-16
Developmental:Test	5.889	1	0.015
Developmental:early_late	85.000	1	< 2.2e-16
Test:early_late	444.746	1	< 2.2e-16
Developmental:Test:early_late	39.164	1	3.896e-10
**June**	**Chisq**	**Df**	**Pr(>Chisq)**
Developmental	3.349	1	0.067
Test	129.720	1	< 2.2e-16
early_late	1732.691	1	< 2.2e-16
Developmental:Test	47.284	1	6.139e-12
Developmental:early_late	76.271	1	< 2.2e-16
Test:early_late	255.286	1	< 2.2e-16
Developmental:Test:early_late	9.254	1	0.002
**July**	**Chisq**	**Df**	**Pr(>Chisq)**
Developmental	30.589	1	3.189e-08
Test	0.977	1	0.322
early_late	1815.515	1	< 2.2e-16
Developmental:Test	222.511	1	< 2.2e-16
Developmental:early_late	26.445	1	2.710e-07
Test:early_late	0.132	1	0.716
Developmental:Test:early_late	127.940	1	< 2.2e-16

Biology Open • Supplementary information

Table S5. Fecundity *horizontal post hoc* tests

Fecundity ETP *horizontal* post hoc test. P- values shown.

ETP	May Early	June Early	July Early	April Late	May Late	June Late	July Late
April Early	<.0001	<.0001	<.0001	0.001	<.0001	0.000	0.001
May Early		<.0001	0.071	<.0001	<.0001	<.0001	<.0001
June Early			0.017	<.0001	<.0001	<.0001	<.0001
July Early				<.0001	<.0001	<.0001	<.0001
April Late					<.0001	<.0001	<.0001
May Late						0.052	0.010
June Late							0.536

Fecundity CON *horizontal post hoc* test. P- values shown.

CON	May Early	June Early	July Early	April Late	May Late	June Late	July Late
April Early	<.0001	<.0001	<.0001	<.0001	<.0001	0.289	0.079
May Early		<.0001	<.0001	<.0001	<.0001	<.0001	<.0001
June Early			<.0001	<.0001	<.0001	<.0001	<.0001
July Early				0.013	<.0001	<.0001	<.0001
April Late					<.0001	<.0001	<.0001
May Late						<.0001	<.0001
June Late							0.005

Biology Open • Supplementary information

Table S6. Developmental time ANOVA

horizontal test.

**ETP**	**Chisq**	**Df**	**Pr(>Chisq)**
Developmental	391.75	3	< 2.2e-16
**CON**	**Chisq**	**Df**	**Pr(>Chisq)**
Developmental	104.02	3	< 2.2e-16

vertical test.

**April**	**Chisq**	**Df**	**Pr(>Chisq)**
Developmental	3.557	1	0.059
Test	324.580	1	< 2e-16
Developmental:Test	5.590	1	0.018
**May**	**Chisq**	**Df**	**Pr(>Chisq)**
Developmental	2.139	1	0.143
Test	4.282	1	0.038
Developmental:Test	0.292	1	0.588
**June**	**Chisq**	**Df**	**Pr(>Chisq)**
Developmental	214.176	1	< 2.2e-16
Test	19.636	1	9.365e-06
Developmental:Test	3.128	1	0.076
**July**	**Chisq**	**Df**	**Pr(>Chisq)**
Developmental	103.179	1	< 2.2e-16
Test	16.782	1	4.192e-05
Developmental:Test	10.659	1	0.001

Biology Open • Supplementary information

Table S7. Developmental time *post hoc* tests

ETP *horizontal post hoc* test. P- values shown.

ETP	May	June	July
April	<.0001	<.0001	<.0001
May		<.0001	<.0001
June			<.0001

CON *horizontal post hoc* test. P- values shown.

CON	May	June	July
April	0.681	<.0001	0.260
May		0.000	0.884
June			<.0001

Vertical post hoc test. Population is listed first, testing temperature second. P- values shown.

**April**	**CONapril in April**	**ETPapril in 25**	**CONapril in 25**
ETPapril in April	0.977	<.0001	<.0001
CONapril in April		<.0001	<.0001
ETPapril in 25			0.002
**July**	**ETPjuly in 25**	**CONjuly in July**	**ETPjuly in July**
CONjuly in 25	<.0001	<.0001	<.0001
ETPjuly in 25		0.000	0.834
CONjuly in July			0.000

Biology Open • Supplementary information

Table S8. Egg-to-adult viability ANOVA

horizontal test.

**ETP**	**Chisq**	**Df**	**Pr(>Chisq)**
Developmental	222.56	3	< 2.2e-16
**CON**	**Chisq**	**Df**	**Pr(>Chisq)**
Developmental	101.82	3	< 2.2e-16

vertical test.

**April**	**Chisq**	**Df**	**Pr(>Chisq)**
Developmental	0.381	1	0.536
Test	28.813	1	7.97e-08
Developmental:Test	6.253	1	0.012
**May**	**Chisq**	**Df**	**Pr(>Chisq)**
Developmental	128.225	1	< 2e-16
Test	2.898	1	0.088
Developmental:Test	0.073	1	0.785
**June**	**Chisq**	**Df**	**Pr(>Chisq)**
Developmental	1.710	1	0.190
Test	152.116	1	< 2.2e-16
Developmental:Test	11.437	1	0.000
**July**	**Chisq**	**Df**	**Pr(>Chisq)**
Developmental	12.997	1	0.000
Test	151.059	1	< 2.2e-16
Developmental:Test	0.267	1	0.604

Biology Open • Supplementary information

Table S9. Egg-to-adult viability *post hoc* tests

ETP *horizontal post hoc* test. P- values shown.

ETP	June	July	May
April	<.0001	<.0001	<.0001
June		<.0001	<.0001
July			<.0001

CON *horizontal post hoc* test. P- values shown.

CON	June	July	May
April	<.0001	<.0001	0.204
June		0.548	0.000
July			<.0001

Vertical post hoc test. P- values shown.

**April**	**CONapril in April**	**ETPapril in 25**	**CONapril in 25**
ETPapril in April	0.209	<.0001	0.000
CONapril in April		<.0001	0.041
ETPapril in 25			0.024
**June**	**ETPjune in 25**	**CONjune in June**	**ETPjune in June**
CONjune in 25	0.004	<.0001	<.0001
ETPjune in 25		<.0001	<.0001
CONjune in June			0.068

Table S10. Adult Survivorship ANOVA

horizontal test.

**ETP**	**Df**	**Chisq**	**Pr(>Chisq)**
Developmental	3	45.119	8.729e-10
sex	1	1.107	0.292
Developmental:sex	3	17.800	0.000
**CON**	**Df**	**Chisq**	**Pr(>Chisq)**
Developmental	3	13.265	0.004
sex	1	5.161	0.023
Developmental:sex	3	9.001	0.029

vertical test.

**April**	**Df**	**Chisq**	**Pr(>Chisq)**
Developmental	1	9.926	0.001
Test	1	65.921	4.693e-16
sex	1	0.026	0.871
Developmental:Test	1	0.397	0.528
Developmental:sex	1	12.548	0.000
Test:sex	1	0.066	0.796
Developmental:Test:sex	1	3.098	0.078
**May**	**Df**	**Chisq**	**Pr(>Chisq)**
Developmental	1	8.893	0.002
Test	1	118.002	< 2.2e-16
sex	1	0.600	0.438
Developmental:Test	1	22.783	1.813e-06
Developmental:sex	1	1.312	0.251
Test:sex	1	2.238	0.134
Developmental:Test:sex	1	9.183	0.002
**June**	**Df**	**Chisq**	**Pr(>Chisq)**
Developmental	1	47.081	6.809e-12
Test	1	59.363	1.311e-14
sex	1	5.012	0.025
Developmental:Test	1	10.590	0.001
Developmental:sex	1	15.153	9.910e-05
Test:sex	1	7.211	0.007
Developmental:Test:sex	1	0.103	0.747
**July**	**Df**	**Chisq**	**Pr(>Chisq)**
Developmental	1	110.979	< 2.2e-16
Test	1	109.482	< 2.2e-16
sex	1	2.087	0.148
Developmental:Test	1	10.068	0.001
Developmental:sex	1	5.401	0.020
Test:sex	1	6.687	0.009

Table S11. Adult Survivorship *post hoc* tests

ETP *horizontal post hoc* test. P- values shown.

ETP	June Female	July Female	May Female	April Male	June Male	July Male	May Male
April Female	<.0001	<.0001	<.0001	0.027	<.0001	<.0001	<.0001
June Female		0.002	0.000	<.0001	0.000	0.000	0.000
July Female			0.676	<.0001	0.471	0.805	0.430
May Female				<.0001	0.766	0.864	0.705
April Male					<.0001	<.0001	<.0001
June Male						0.638	0.938
July Male							0.592

CON *horizontal post hoc* test. P- values shown.

CON	June Female	July Female	May Female	April Male	June Male	July Male	May Male
April Female	<.0001	0.056	0.001	0.428	0.063	0.078	0.602
June Female		0.019	0.321	0.000	0.016	0.012	0.000
July Female			0.185	0.246	0.946	0.873	0.157
May Female				0.012	0.159	0.135	0.005
April Male					0.272	0.315	0.787
June Male						0.926	0.170
July Male							0.204

Biology Open • Supplementary information

Table S11. Adult survivorship *post hoc* tests (continued)

May *vertical post hoc* test. P- values shown.

May	ETPmay in 25 Female	CONmay in May Female	ETPmay in May Female	CONmay in 25 Male	ETPmay in 25 Male	CONmay in May Male	ETPmay in May Male
CONmay in 25 Female	0.003	<.0001	<.0001	0.013	0.235	<.0001	<.0001
ETPmay in 25 Female		<.0001	<.0001	0.599	0.077	<.0001	<.0001
CONmay in May Female			<.0001	<.0001	<.0001	0.034	<.0001
ETP in May Female				<.0001	<.0001	0.003	0.748
CONmay in 25 Male					0.203	<.0001	<.0001
ETPmay in 25 Male						<.0001	<.0001
CONmay in May Male							0.001

Biology Open • Supplementary information

Table S11. Adult survivorship *post hoc* tests (continued)

June *vertical dev*sex post hoc* test. P- values shown.

June	ETPjune Female	CONjune Male	ETPjune Male
CONjune Female	0.009	0.277	<.0001
ETPjune Female		0.000	<.0001
CONjune Male			<.0001

July *vertical post hoc* test. P- values shown.

July	ETPjuly in 25 Female	CONjuly in July Female	ETPjuly in July Female	CONjuly in 25 Male	ETPjuly in 25 Male	CONjuly in July Male	ETPjuly in July Male
CONjuly in 25 Female	<.0001	0.000	<.0001	0.519	<.0001	<.0001	<.0001
ETPjuly in 25 Female		0.175	<.0001	<.0001	0.571	0.001	<.0001
CONjuly in July Female			<.0001	0.000	0.430	<.0001	<.0001
ETPjuly in July Female				<.0001	<.0001	<.0001	0.738
CONjuly in 25 Male					<.0001	<.0001	<.0001
ETPjuly in 25 Male						0.000	<.0001
CONjuly in July Male							<.0001

Biology Open • Supplementary information

Table S12. Adult heat stress ANOVA

horizontal test.

**ETP**	**LR Chisq**	**Df**	**Pr(>Chisq)**
Developmental	208.285	3	< 2.2e-16
Sex	18.522	1	1.68e-05
Developmental:Sex	8.547	3	0.035
**CON**	**LR Chisq**	**Df**	**Pr(>Chisq)**
Developmental	16.669	3	0.000
Sex	1.048	1	0.305
Developmental:Sex	11.733	3	0.008

vertical test.

**April**	**LR Chisq**	**Df**	**Pr(>Chisq)**
Developmental	5.684	1	0.017
Sex	3.160	1	0.075
Developmental:Sex	1.115	1	0.290
**May**	**LR Chisq**	**Df**	**Pr(>Chisq)**
Developmental	40.828	1	1.662e-10
Sex	9.036	1	0.002
Developmental:Sex	0.650	1	0.420
**June**	**LR Chisq**	**Df**	**Pr(>Chisq)**
Developmental	123.471	1	<2e-16
Sex	3.637	1	0.056
Developmental:Sex	0.042	1	0.836
**July**	**LR Chisq**	**Df**	**Pr(>Chisq)**
Developmental	124.033	1	< 2.2e-16
Sex	0.585	1	0.444
Developmental:Sex	8.971	1	0.002

Biology Open • Supplementary information

Table S13. Adult heat stress *post hoc* tests

ETP *horizontal post hoc* test. P- values shown.

ETP	July Female	June Female	May Female	April Male	July Male	June Male	May Male
April Female	<.0001	<.0001	<.0001	0.015	<.0001	<.0001	0.000
July Female		0.624	<.0001	<.0001	0.261	0.462	<.0001
June Female			<.0001	<.0001	0.108	0.221	<.0001
May Female				<.0001	<.0001	<.0001	<.0001
April Male					<.0001	<.0001	<.0001
July Male						0.698	<.0001
June Male							<.0001

CON *horizontal post hoc* test. P- values shown.

CON	June Female	July Female	May Female	April Male	June Male	July Male	May Male
April Female	0.201	0.465	0.353	0.587	0.939	0.123	0.000
June Female		0.042	0.028	0.063	0.228	0.765	<.0001
July Female			0.835	0.841	0.416	0.023	0.005
May Female				0.682	0.307	0.014	0.011
April Male					0.532	0.034	0.003
June Male						0.140	0.000
July Male							<.0001

Biology Open • Supplementary information

Table S14. Larval heat stress ANOVA

horizontal test.

**ETP**	**LR Chisq**	**Df**	**Pr(>Chisq)**
Developmental	47.668	3	2.505e-10
**CON**	**LR Chisq**	**Df**	**Pr(>Chisq)**
Developmental	19.178	3	0.0001

vertical test.

**April**	**LR Chisq**	**Df**	**Pr(>Chisq)**
Developmental	32.089	1	1.473e-08
**May**	**LR Chisq**	**Df**	**Pr(>Chisq)**
Developmental	39.403	1	3.448e-10
**June**	**LR Chisq**	**Df**	**Pr(>Chisq)**
Developmental	8.054	1	0.004
**July**	**LR Chisq**	**Df**	**Pr(>Chisq)**
Developmental	13.044	1	0.0001

Table S15. Larval heat stress *horizontal post hoc* test. P- values shown.

**ETP**	**June**	**July**	**May**
AP	<.0001	<.0001	<.0001
JU		0.0537	.7963
L			0.389
**CON**	**June**	**July**	**May**
April	0.002	0.577	0.441
June		<.0001	<0001
July			0.834

Biology Open • Supplementary information

Table S16. Desiccation and nutritional stress ANOVA

Desiccation *vertical* test

June	LR Chisq	Df	Pr(>Chisq)
Developmental	49.040	1	2.507e-12
sex	0.103	1	0.747
Developmental:sex	4.231	1	0.039
**July**	**LR Chisq**	**Df**	**Pr(>Chisq)**
Developmental	13.386	1	0.000
Test	82.536	1	< 2.2e-16
sex	16.821	1	4.107e-05
Developmental:Test	1.946	1	0.163
Developmental:sex	15.395	1	8.723e-05
Test:sex	0.883	1	0.347
Developmental:Test:sex	0.725	1	0.394

Starvation *vertical* test

July	LR Chisq	Df	Pr(>Chisq)
Developmental	27.767	1	1.369e-07
Test	107.177	1	< 2.2e-16
sex	20.440	1	6.154e-06
Developmental:Test	2.847	1	0.091
Developmental:sex	10.212	1	0.001
Test:sex	2.811	1	0.093
Developmental:Test:sex	0.000	1	0.998

Table S17. Desiccation *vertical post hoc* tests. P- values shown.

June	ETPjune Female	CONjune Male	ETPjune Male
CONjun Female	<.0001	0.216	<.0001
ETPjune Female		<.0001	0.091
CONjune Male			<.0001
**July**	**ETPjuly Female**	**CONjuly Male**	**ETPjuly Male**
CONjuly Female	<.0001	0.684	0.955
ETPjuly Female		<.0001	<.0001
CONjuly Male			0.729

Biology Open • Supplementary information

Table S18. Metabolic pools ANOVA

Dry weight ANOVA *horizontal* test

**ETP**	**Sum Sq**	**Df**	**F value**	**Pr(>F)**
Developmental	6.473	3	89.335	1.23e-15
Sex	7.890	1	326.699	< 2.2e-16
Developmental:Sex	0.459	3	6.336	0.001
Residuals	0.772	32		
**CON**	**Sum Sq**	**Df**	**F value**	**Pr(>F)**
Developmental	1.875	3	41.693	3.654e-11
Sex	8.021	1	534.969	< 2.2e-16
Developmental:Sex	0.496	3	11.038	3.918e-05
Residuals	0.479	32		

Dry weight ANOVA *vertical* test

**April**	**Sum Sq**	**Df**	**F value**	**Pr(>F)**
Developmental	0.005	1	0.407	0.532
Sex	7.478	1	526.692	1.136e-13
Developmental:Sex	0.008	1	0.574	0.459
Residuals	0.227	16		
**May**	**Sum Sq**	**Df**	**F value**	**Pr(>F)**
Developmental	0.045	1	2.173	0.159
Sex	2.712	1	129.794	4.349e-09
Developmental:Sex	0.020	1	0.983	0.336
Residuals	0.334	16		
**June**	**Sum Sq**	**Df**	**F value**	**Pr(>F)**
Developmental	0.008	1	0.901	0.356
Sex	3.843	1	390.700	1.147e-12
Developmental:Sex	0.043	1	4.368	0.052
Residuals	0.157	16		
**July**	**Sum Sq**	**Df**	**F value**	**Pr(>F)**
Developmental	2.203	1	66.052	4.516e-07
Sex	2.676	1	80.231	1.242e-07
Developmental:Sex	0.085	1	2.575	0.128
Residuals	0.533	16		

Biology Open • Supplementary information

Table S18. Metabolic pools ANOVA (continued)

Glycogen ANOVA *horizontal* test

**ETP *horizontal***	**Sum Sq**	**Df**	**F value**	**Pr(>F)**
Developmental	0.076	3	7.734	0.000
sex	0.015	1	4.601	0.039
Developmental:sex	0.031	3	3.166	0.037
Residuals	0.104	32		
**CON *horizontal***	**Sum Sq**	**Df**	**F value**	**Pr(>F)**
Developmental	0.068	3	23.960	2.558e-08
sex	0.057	1	60.064	7.746e-09
Developmental:sex	0.000	3	0.252	0.858
Residuals	0.030	32		

Glycogen ANOVA *vertical* test

**April**	**Sum Sq**	**Df**	**F value**	**Pr(>F)**
Developmental	0.075	1	30.967	4.27e-05
sex	0.036	1	15.011	0.001
Developmental:sex	0.002	1	0.839	0.373
Residuals	0.038	16		
**May**	**Sum Sq**	**Df**	**F value**	**Pr(>F)**
Developmental	0.029	1	22.525	0.0001
sex	0.030	1	23.759	0.0001
Developmental:sex	0.000	1	0.084	0.775
Residuals	0.020	16		
**June**	**Sum Sq**	**Df**	**F value**	**Pr(>F)**
Developmental	0.070	1	47.498	3.624e-06
sex	0.008	1	6.011	0.026
Developmental:sex	0.003	1	2.403	0.140
Residuals	0.023	16		
**July**	**Sum Sq**	**Df**	**F value**	**Pr(>F)**
Developmental	0.013	1	4.326	0.053
sex	0.002	1	0.770	0.393
Developmental:sex	0.019	1	5.990	0.026
Residuals	0.051	16		

Biology Open • Supplementary information

Table S18. Metabolic pools ANOVA (continued)

Triglyceride ANOVA *horizontal* test

**ETP**	**Sum Sq**	**Df**	**F value**	**Pr(>F)**
Developmental	0.012	3	4.680	0.008
sex	0.069	1	80.585	2.967e-10
Developmental:sex	0.005	3	2.233	0.103
Residuals	0.027	32		
**CON**	**Sum Sq**	**Df**	**F value**	**Pr(>F)**
Developmental	0.017	3	6.076	0.002
sex	0.046	1	48.606	6.734e-08
Developmental:sex	0.003	3	1.245	0.309
Residuals	0.030	32		

Triglyceride ANOVA *vertical* test

**April**	**Sum Sq**	**Df**	**F value**	**Pr(>F)**
Developmental	0.005	1	13.038	0.002
sex	0.033	1	75.751	1.827e-07
Developmental:sex	0.000	1	2.111	0.165
Residuals	0.007	16		
**May**	**Sum Sq**	**Df**	**F value**	**Pr(>F)**
Developmental	0.000	1	0.000	0.997
sex	0.029	1	25.929	0.000
Developmental:sex	0.000	1	0.001	0.970
Residuals	0.018	16		
**June**	**Sum Sq**	**Df**	**F value**	**Pr(>F)**
Developmental	0.011	1	15.275	0.001
sex	0.015	1	21.184	0.0001
Developmental:sex	0.000	1	0.303	0.589
Residuals	0.011	16		
**July**	**Sum Sq**	**Df**	**F value**	**Pr(>F)**
Developmental	0.032	1	24.938	0.000
sex	0.038	1	29.532	5.511e-05
Developmental:sex	0.006	1	4.864	0.042
Residuals	0.020	16		

Biology Open • Supplementary information

Table S18. Metabolic pools ANOVA (continued)

Protein ANOVA *horizontal* test

**ETP**	**Sum Sq**	**Df**	**F value**	**Pr(>F)**
Developmental	0.031	3	12.716	1.226e-05
sex	0.000	1	1.079	0.306
Developmental:sex	0.007	3	3.083	0.041
Residuals	0.026	32		
**CON**	**Sum Sq**	**Df**	**F value**	**Pr(>F)**
Developmental	0.025	3	10.595	5.402e-05
sex	0.014	1	17.553	0.000
Developmental:sex	0.002	3	1.132	0.350
Residuals	0.025	32		

Protein ANOVA *vertical* test

**April**	**Sum Sq**	**Df**	**F value**	**Pr(>F)**
Developmental	0.000	1	0.221	0.644
sex	0.004	1	3.975	0.063
Developmental:sex	0.004	1	3.991	0.063
Residuals	0.018	16		
**May**	**Sum Sq**	**Df**	**F value**	**Pr(>F)**
Developmental	0.000	1	0.048	0.829
sex	0.006	1	11.264	0.004
Developmental:sex	0.000	1	0.235	0.634
Residuals	0.009	16		
**June**	**Sum Sq**	**Df**	**F value**	**Pr(>F)**
Developmental	0.000	1	0.109	0.745
sex	0.000	1	0.016	0.899
Developmental:sex	0.005	1	5.871	0.027
Residuals	0.014	16		
**July**	**Sum Sq**	**Df**	**F value**	**Pr(>F)**
Developmental	0.001	1	2.650	0.123
sex	0.000	1	1.174	0.294
Developmental:sex	0.003	1	5.582	0.031
Residuals	0.009	16		

Biology Open • Supplementary information

Table S19. Metabolic pools *post hoc* tests

Glycogen *horizontal* post hoc tests. P- values shown

**ETP**	**June**	**July**	**May**
April	0.303	0.007	0.133
June		0.000	0.623
July			0.000
**CON**	**June**	**July**	**May**
April	0.032	<.0001	<.0001
June		<.0001	0.000
July			0.230

Glycogen July *vertical* post hoc test. P- values shown

	ETPjuly female	CONjuly male	ETPjuly male
CONjuly female	0.798	0.031	0.407
ETPjuly female		0.052	0.283
CONjuly male			0.005

Triglyceride *horizontal* post hoc tests. P- values shown

**ETP**	**June**	**July**	**May**
April	0.077	0.129	0.169
June		0.001	0.680
July			0.005
**CON**	**June**	**July**	**May**
April	0.011	0.066	0.268
June		0.433	0.000
July			0.004

Triglyceride July *vertical* post hoc test. P- values shown

	ETPjuly female	CONjuly male	ETPjuly male
CONjuly female	0.066	0.036	<.0001
ETPjuly female		0.759	<.0001
CONjuly male			0.0006

Biology Open • Supplementary information

Table S19. Metabolic pools *post hoc* tests (continued)

Protein ETP *horizontal* post hoc test. P- values shown

ETP	June	July	May
April	0.034	0.000	0.080
June		0.088	0.000
July			<.0001

Protein *vertical* post hoc tests. P- values shown

**June**	**ETPjune female**	**CONjune male**	**ETPjune male**
CONjune female	0.069	0.090	0.749
ETPjune female		0.887	0.124
CONjune male			0.158
**July**	**ETPjuly female**	**CONjuly male**	**ETPjuly male**
CONjuly female	0.610	0.379	0.073
ETPjuly female		0.705	0.026
CONjuly male			0.012

Biology Open • Supplementary information

Table S20. Metabolic pools female only *post hoc* tests

Female glycogen post hoc tests. P- values shown

	CONapril	CONjune	CONjuly	CONmay	ETPjune	ETPjuly	ETPmay
ETPapril	0.0002	0.0045	0.693	0.291	0.0054	0.992	0.0280
CONapril		0.237	0.0005	0.0032	<.0001	0.0002	<.0001
CONjune			0.0121	0.0556	<.0001	0.0046	<.0001
CONjuly				0.505	0.0019	0.7001	0.0110
CONmay					0.0003	0.296	0.0019
ETPjune						0.0052	0.4980
ETPjuly							0.0273

Female triglyceride post hoc tests. P- values shown

	CONapril	CONjune	CONjuly	CONmay	ETPjune	ETPjuly	ETPmay
ETPapril	0.0077	0.0008	0.0038	0.187	0.218	0.668	0.176
CONapril		0.404	0.7840	0.144	0.122	0.0217	0.154
CONjune			0.573	0.0255	0.0206	0.0027	0.0277
CONjuly				0.0857	0.0713	0.0113	0.092
CONmay					0.927	0.367	0.971
ETPjune						0.416	0.898
ETPjuly							0.348

Female protein post hoc tests. P- values shown

	CONapril	CONjune	CONjuly	CONmay	ETPjune	ETPjuly	ETPmay
ETPapril	0.162	0.0040	0.0188	0.785	0.435	0.0571	0.652
CONapril		0.105	0.305	0.0974	0.526	0.592	0.0682
CONjune			0.536	0.0019	0.0276	0.269	0.0012
CONjuly				0.0097	0.102	0.620	0.0062
CONmay					0.294	0.0315	0.858
ETPjune						0.246	0.222
ETPjuly							0.0209

Female dry weight post hoc tests. P- values shown

	CONapril	CONjune	CONjuly	CONmay	ETPjune	ETPjuly	ETPmay
ETPapril	0.948	<.0001	<.0001	<.0001	<.0001	<.0001	<.0001
CONapril		<.0001	<.0001	<.0001	<.0001	<.0001	<.0001
CONjune			0.0364	0.284	0.176	<.0001	0.0103
CONjuly				0.283	0.430	<.0001	0.590
CONmay					0.771	<.0001	0.111
ETPjune						<.0001	0.189
ETPjuly							<.0001

Biology Open • Supplementary information

Table S21. Metabolic pools male only *post hoc* tests

Male glycogen post hoc tests. P- values shown

	CONapril	CONjune	CONjuly	CONmay	ETPjune	ETPjuly	ETPmay
ETPapril	0.0002	0.0023	0.362	0.157	0.586	0.0001	0.485
CONapril		0.335	0.0020	0.0077	0.0007	0.933	<.0001
CONjune			0.0231	0.0717	0.0095	0.295	0.0003
CONjuly				0.604	0.710	0.0016	0.113
CONmay					0.376	0.0062	0.0388
ETPjune						0.0006	0.218
ETPjuly							<.0001

Male triglyceride post hoc tests. P- values shown

	CONapril	CONjune	CONjuly	CONmay	ETPjune	ETPjuly	ETPmay
ETPapril	0.344	0.0006	0.0028	0.508	0.217	0.0302	0.524
CONapril		0.0073	0.0298	0.772	0.766	0.0029	0.753
CONjune			0.559	0.0035	0.0153	<.0001	0.0033
CONjuly				0.0152	0.0571	<.0001	0.0143
CONmay					0.558	0.0061	0.980
ETPjune						0.0013	0.542
ETPjuly							0.0065

Male protein post hoc tests. P- values shown

	CONapril	CONjune	CONjuly	CONmay	ETPjune	ETPjuly	ETPmay
ETPapril	0.0684	0.441	0.202	0.0252	0.0344	0.0013	0.0588
CONapril		0.012	0.0032	0.647	0.0003	<.0001	0.942
CONjune			0.604	0.0037	0.163	0.0100	0.0100
CONjuly				0.0009	0.372	0.0339	0.0026
CONmay					<.0001	<.0001	0.700
ETPjune						0.200	0.0002
ETPjuly							<.0001

Male dry weight post hoc tests. P- values shown

	CONapril	CONjune	CONjuly	CONmay	ETPjune	ETPjuly	ETPmay
ETPapril	0.351	<.0001	0.0009	0.0146	<.0001	<.0001	0.0363
CONapril		0.0001	0.0109	0.112	<.0001	<.0001	0.224
CONjune			0.0985	0.0093	0.525	<.0001	0.0034
CONjuly				0.293	0.0254	<.0001	0.153
CONmay					0.0018	<.0001	0.694
ETPjune						0.0001	0.0006
ETPjuly							<.0001

Biology Open • Supplementary information
